# Prospective malaria control using entomopathogenic fungi: comparative evaluation of impact on transmission and selection for resistance

**DOI:** 10.1186/1475-2875-11-383

**Published:** 2012-11-22

**Authors:** Penelope A Lynch, Uwe Grimm, Matthew B Thomas, Andrew F Read

**Affiliations:** 1 Mathematics and Statistics Department, The Faculty of Mathematics, Computing and Technology, The Open University, Walton Hall, Milton Keynes, UK; 2 Center for Infectious Disease Dynamics, Department of Entomology, Pennsylvania State University, University Park, Pennsylvania, USA; 3 Center for Infectious Disease Dynamics, Departments of Biology and Entomology, Pennsylvania State University, University Park, Pennsylvania, USA; 4 Fogarty International Center, National Institutes of Health, Bethesda, Maryland, USA

## Abstract

**Background:**

Chemical insecticides against adult mosquitoes are a key element in most malaria management programmes, but their efficacy is threatened by the evolution of insecticide-resistant mosquitoes. By killing only older mosquitoes, entomopathogenic fungi can in principle significantly impact parasite transmission while imposing much less selection for resistance. Here an assessment is made as to which of the wide range of possible virulence characteristics for fungal biopesticides best realise this potential.

**Methods:**

With mathematical models that capture relevant timings and survival probabilities within successive feeding cycles, transmission and resistance-management metrics are used to compare susceptible and resistant mosquitoes exposed to no intervention, to conventional instant-kill interventions, and to delayed-action biopesticides with a wide range of virulence characteristics.

**Results:**

Fungal biopesticides that generate high rates of mortality at around the time mosquitoes first become able to transmit the malaria parasite offer potential for large reductions in transmission while imposing low fitness costs. The best combinations of control and resistance management are generally accessed at high levels of coverage. Strains which have high virulence in malaria-infected mosquitoes but lower virulence in malaria-free mosquitoes offer the ultimate benefit in terms of minimizing selection pressure whilst maximizing impact on transmission. Exploiting this phenotype should be a target for product development. For indoor residual spray programmes, biopesticides may offer substantial advantages over the widely used pyrethroid-based insecticides. Not only do fungal biopesticides provide substantial resistance management gains in the long term, they may also provide greater reductions in transmission before resistance has evolved. This is because fungal spores do not have contact irritancy, reducing the chances that a blood-fed mosquito can survive an encounter and thus live long enough to transmit malaria.

**Conclusions:**

Delayed-action products, such as fungal biopesticides, have the potential to achieve reductions in transmission comparable with those achieved with existing instant-kill insecticides, and to sustain this control for substantially longer once resistant alleles arise. Given the current insecticide resistance crisis, efforts should continue to fully explore the operational feasibility of this alternative approach.

## Background

The impressive reductions in global malaria burden achieved this century by chemical insecticides against adult mosquitoes could be eroded by insecticide-resistant mosquitoes [1-6], just as they were last century [7]. In principle, the evolution of insecticide resistance could be considerably slowed and perhaps prevented altogether by vector control aimed at killing only older mosquitoes, so-called late-life action (LLA) [8]. Malaria parasites in a mosquito host take at least nine days to develop to a stage which can be transmitted to a human via an infectious bite [9]. Since mortality in wild mosquito populations is high, the majority of eggs are produced by young mosquitoes. Thus, a vector-control treatment which kills only older mosquitoes could remove infected mosquitoes before they can transmit malaria whilst only impacting the reproductive success of only the relatively few mosquitoes that survive to old age. This would dramatically reduce transmission while exerting only weak selection for resistance.

One option for an LLA vector-control measure is entomopathogenic fungi [10]. Naturally occurring strains of two fungi, *Beauveria bassiana* and *Metarhizium anisopliae*, are already in commercial use for agricultural applications and have been shown to infect and kill mosquitoes in laboratory and field settings. Fungal spores can be picked up by mosquitoes following contact with treated surfaces, and so could be used against mosquitoes in indoor residual spray (IRS) programmes, or delivered via traps, curtains or netting [11-16].

A wide variety of mortality schedules can be induced in *Anopheles* by entomopathogenic fungi [17]. In some cases, all mosquitoes can be killed within a few days; in others, background mortality rates can be barely altered. This virulence variation depends on isolate [11], dose [18] and malaria-infection status [15,18], see also [19]. Lethality can also be increased by genetically modifying fungal isolates [20-22].

If fungal entomopathogens are to realize the potential of the LLA approach to sustainable malaria control, candidate biopesticides need to be chosen which balance reductions in parasite transmission (maximized by high fungal virulence) with resistance management (maximized by low fungal virulence). Here a mathematical model is used to ask which virulence phenotypes best achieve this balance. The intention is to guide the development of target product profiles. The possible efficacy of fungal biopesticides in IRS campaigns is compared with that of pyrethroid-based insecticides now in widespread use. Pyrethroids are highly lethal if contacted by a mosquito, but they also have a strong excito-repellency effect, which can drive away mosquitoes before they receive a lethal dose [23-25]. There is evidence that fungal spores do not repel mosquitoes [26], raising the prospect that, for IRS, fungal biopesticides might more effectively reduce transmission than pyrethroid-based technologies currently in use.

## Methods

### The model

Many malaria transmission models already exist [27], but most do not capture the detailed timings and probabilities of infection, infectiousness, reproduction and mortality over the mosquito lifespan which are key to assessing whether LLAs can provide a useful balance of transmission control and low selection for resistance. In order to encompass these elements, a model has been developed with two separate components, a markovian, deterministic, feeding cycle model (FCM) which calculates survival, egg-laying and infectious bite values during the lifetime of an adult mosquito, and a population model (PM) which tracks the population-level spread of resistance alleles and corresponding loss of transmission control. The model is a development of a simpler version previously used to evaluate putative chemical LLAs [8]. Other modelling frameworks used to assess the LLA approach are heuristically useful but lack sufficient detail to define target virulence schedules [28-30].

### The feeding cycle model

The FCM calculates survival, egg-laying and infectious bite values across a series of discrete adult age classes for a specified type of mosquito (e.g., susceptible) subjected to a given intervention (e.g., a particular fungal biopesticide at a particular coverage). Each sequential age class is defined as lasting for the average length of one gonotrophic cycle. Use of the mosquito feeding cycle as the basis for age-structured analyses of mosquito populations is well established [31-34].

**Figure 1:**
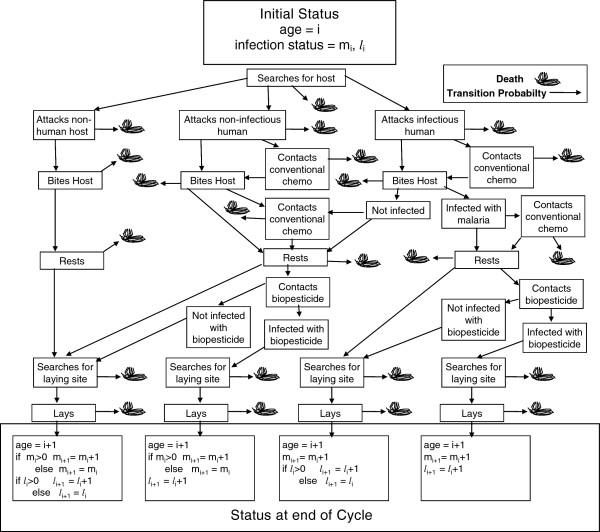
**FCM model structure for one feeding cycle.** Calculation of possible outcomes for feeding cycle *i*+1 of the model, assuming status when commencing cycle *i*+1 is age = *i*, malaria status = *m*_*i*_, fungal infection status = *l*_*i*_, with *l*_0_=0 and *m*_0_=0. Each arrow represents a probability calculated by the model. If the malaria infection status (number of cycles since infection) of a mosquito when biting is greater than the development time of the malaria parasite in the mosquito, then ‘bites host’ after attacking a human host is recorded as an infectious bite.

The FCM tracks possible states and transitions through each age class (*i*), applying survival, exposure and infection probabilities (Figure 1). Infection status for a biopesticide (*l*) or malaria (*m*), is zero for no infection, otherwise equal to the age of the infection. State changes depend on the preceding state, the passage of time, mortality rates and the probabilities of certain events, such as contacting a biopesticide when resting after a human blood meal. For example, for a case analysing the effects of a fungal biopesticide, a mosquito commencing its fourth cycle with an infectious, three-cycle-old malaria infection, and no fungal infection, will spend a defined period of time searching for a host, with an associated probability of dying from background mortality while it does so. It will then attack a host, with a given probability that the selected host will be a non-infectious human, a malaria-infectious human, or non-human, and a given probability of being killed whilst attacking the host before biting. If it survives to bite, and if the host is human, this is recorded as an infectious bite. There is then a given probability that it is killed by the host after biting. If it is not killed, it begins a period of resting, during which, if the chosen host was human, it has a fixed probability of encountering and being infected by the fungus, as well as a given probability of dying from background mortality before leaving to search for an egg-laying site. During the search for an egg-laying site there is a given probability that the mosquito may die from background mortality, or from the effects of its newly acquired fungal infection. If it survives searching for an egg-laying site it may die before or after laying with given probabilities. If still alive at the end of the cycle, it begins its fifth cycle with an infectious, four-cycle-old malaria infection, and a one-cycle-old fungal infection. For a case analysing the effects of a conventional instant-kill chemical pesticide, the analysis would include a probability of contacting the pesticide after biting the host, and a probability of death, assumed to be instant, resulting from that contact, with zero probability of contacting a biopesticide.

Both conventional instant-kill and delayed-action biopesticides offer public health benefits by reducing the numbers of mosquitoes that survive to give infectious bites in a treated population. Clearly the extent to which a reduction in infectious bites maps to reduced transmission and reduced numbers or severity of malaria cases in a human host population involves many complex, context-specific factors. For comparative purposes, however, it is assumed that in a given context, a given reduction in infectious bites will generate the same reduction in malaria transmission and malaria morbidity and mortality irrespective of the type of intervention from which it results. For generality, therefore, the comparative public health benefits of the insecticides considered in this analysis are all evaluated based on the reduction in infectious bites which they provide. This is quantified in the FCM for a given phenotype by calculating RAIB, the proportionate reduction in the average number of infectious bites per mosquito per lifetime (AIB), defined as

$$\text{RAIB}=1-\frac{\text{AIB with treatment}}{\text{AIB without treatment}}$$

Assuming that the rate at which newly maturing adults join a population is constant through time, and that the size of the human host population is unaffected by the intervention being assessed, RAIB is equal to the proportionate reduction in the entomological inoculation rate (EIR), the number of infectious bites experienced per person per unit of time.

To evaluate mosquito fitness, the average number of eggs produced per mosquito per lifetime is used as a proxy for lifetime reproductive success (LRS). The selection coefficient, the proportionate fitness benefit of resistance to a given intervention, is calculated as $\text{Selection Coefficient}=1-\frac{\text{LRS for specified mosquito type with intervention}}{\text{LRS for susceptible mosquitoes without intervention}}$. A selection coefficient of zero means no selection pressure in favour of resistance, with higher selection coefficients indicating increasingly strong selection for resistance.

Formulating these key variables in relative terms minimizes the sensitivity of the conclusions to parameter values that are independent of the vector-control treatment or mosquito phenotype being evaluated.

The primary definitions of the FCM are given in Table 1, and its main features are detailed below. A detailed derivation of the FCM is given in Additional file 1: Appendix A. Baseline parameter values used in the analysis are summarized in Table 2.

**Table 1:** Variables and parameters for the feeding cycle model

**Variable or Parameter**	**Symbol**	**Comments and Constraints**
Time, measured in whole units equal to average length of sporogonic cycle, from infection of mosquito by malaria to cycle from which mosquito gives infectious bites	*D*	*input* 0<*D*
Number of age classes included in analysis	*λ*	
Cycle number *(identifies specific cycle in the λ cycles over which probabilities are tracked in the FCM)*	*i*	0*≤ i ≤λ*
Malaria status, the number of whole or partial cycles since infection with malaria	*m*	0*≤ m ≤λ, m* = 0 *means not infected*
Biopesticide infection status, the number of whole or partial cycles since infection with biopesticide	*l*	0*≤ l≤ λ*, *l* = 0 *means not infected*
Average number of eggs laid in cycle *i* by mosquitoes surviving to the start of cycle *i*	*F*_*i*_	
Average lifetime number of eggs laid per mosquito	*φ*	
Average number of eggs laid in cycle *i*, by mosquitoes starting cycle *i* with malaria status *m* and biopesticide status *l*	*f*_*i,m,l*_	*m*<*i l*<*i*
Average probability of survival from start of cycle *i* to start of cycle *i*+1	*S*_*i*_	
Average probability that a mosquito starting cycle *i* with malaria status *m* and biopesticide status *l,* will survive to start of cycle *i*+1	*s*_*i,m,l*_	*m*<*i l*<*i*
Average probability of a mosquito being alive at start of cycle *i.*	*V*_*i*_	
Average probability of a mosquito being alive, with malaria status *m* and biopesticide status *l*, at start of period *i.*	*v*_*i,m,l*_	*m*<*i l*<*i*
Probability that a mosquito alive at start of cycle *i* with malaria status *m* and biopesticide status *l*, survives and bites host type *h* in cycle *i*	*q*_*i,m,l,h*_	*m*<*i l*<*i*
Type of host attacked	*h*	*h*=1, non-human
		*h*=2, non-infectious human
		*h*=3, infectious human
Average number of infectious bites in cycle *i* per mosquito alive at the start of cycle *i*	*I*_*i*_	
Average lifetime number of infectious bites per mosquito	*u*	

**Table 2:** Values used in FCM for this analysis

**Variable or Parameter**	**Symbol**	**Value**	***units***
Background instantaneous mortality rate for mosquito age *i*	*r*_*B,i*_	**11.75%**^**1**^	*per day*
Length of gonotrophic cycle	*w*	**2.85**^**1**^	*days*
Time spent host searching and feeding during a cycle	*b*	**1.26**^**5**^	*days*
Time spent finding oviposition site and laying during a cycle	*ϕ*	**1.26**^**5**^	*days*
Length of resting period (days)	*η*	**0.32**^**5**^	*days*
Proportion human population infectious for malaria^4^	*p*	**4.28%**^**1**^	
Probability attacks non-human host	*H*	**0.17**^**1**^	
Probability killed when attacking host before biting	*a*_*1*_	**.05**^**6**^	
Probability killed when attacking host after biting (excluding mortality from insecticide treatments)	*a*_*2*_	**.05**^**6**^	
Probability becomes infected with malaria when biting infectious human host^4^	*M*	**1.00**	
Number of eggs laid per successfully laying mosquito per cycle	*L*	**100**^**2**^	*eggs*
Time, measured in whole units equal to length of gonotrophic cycle, from infection of mosquito to cycle from which mosquito gives infectious bites	*D*	**3**^**3**^ Based on 10.78 ^1^ days	*cycles*
Baseline probability that mosquito contacts and is killed by conventional instant-kill chemical insecticide (CC) whilst resting after biting human host	*k*	**0***for cases not assessing use of CC*	
		**0.8***for cases assessing use of CC*	
Baseline probability that mosquito contacts and is affected by delayed action pesticide whilst resting after biting human host	*X*	**0***for cases not assessing use of delayed action pesticide*	
		**0.8***for cases assessing use of delayed action pesticide*	
Number of age classes included in analysis	*λ*	**10**	*cycles*

*1.Averages taken from four geographic locations *[31]*. Results using individual geographic data sets are expected to give qualitatively equivalent results. Limited sensitivity analysis was consistent with this assumption, so use of the average figures was considered adequate for the present analysis.*

2.Since we are only interested in comparative values, the absolute value for the number of eggs per lay is immaterial, 100 has been used as a convenient normalised value.

3.The number of cycles assumed for sporogonic development is calculated from the average number of days for sporogonic development and the average number of days per gonotrophic cycle, rounded down to give a whole number of cycles. This is a conservative assumption with respect to the amount of EIR reduction calculated for given fungal virulence parameters.

4.The data set used provides a total probability of acquiring a malaria infection when biting a human host. This has been used as the value for parameter p, with M=1.00, to give the appropriate combined probability, Mp.

5. Assumes c.11.1% of every cycle is spent resting (8 hours in a 72 hour cycle), with the rest of the gonotrophic cycle divided equally between laying and feeding.

*6. Estimated 10% mortality per feeding attempt*[35]*, divided equally between pre- and post-bite.*

The probability that a mosquito contacts and is affected (killed or infected) by a conventional or biological insecticide after biting a human host is input as a single ‘coverage’ value, incorporating the probabilities of being in a treated property, of contacting the pesticide, and of being affected by the pesticide during contact. It is assumed that physical constraints on the proportion of surfaces and internal areas treated will apply equally to conventional and fungal insecticides, and that for mosquitoes contacting treated surfaces, biopesticides can potentially offer rates of infection equivalent to the rates of mortality generated by conventional insecticides. The latter assumption is supported by field trials showing >86% infection of mosquitoes entering outdoor bait boxes [36], 76% infection in experimental huts with fungus-impregnated eave curtains [13], and laboratory trials showing >95% infection from treated clay pots [14] or exposure to treated clay tiles [11].

The average number of eggs laid in a given cycle, by mosquitoes surviving to the start of that cycle, *F*_*i*_, is calculated as

$$F_{i}=\frac{\left(\sum_{m=0}^{i-1}\sum_{l=0}^{i-1}f_{i,m,l}v_{i,m,l}\right)}{V_{i}}$$

This provides the basis for the evaluation of relative fitness using a comparison of values for *φ*, lifetime egg production, representing LRS, $\varphi =\sum_{i=1}^{\lambda }F_{i}V_{i}$.

Comparative levels of transmission control are assessed using *u*, the average number of infectious bites per mosquito lifetime, $u=\sum_{i=1}^{\lambda }I_{i}V_{i}$.

The average number of infectious bites during cycle *i* per mosquito surviving to the beginning of cycle *i*, *I*_*i*_, is calculated as

$I_{i}=\frac{\sum_{m=D}^{i-1}\sum_{l=0}^{i-1}q_{i,m,l,2}v_{i,m,l}+q_{i,m,l,3}v_{i,m,l}}{V_{i}}$*i* >*D*

The average probability of survival from start of cycle *i* to start of cycle (*i* + 1) is *S*_*i*_, with

$$S_{i}=\frac{\left(\sum_{m=0}^{i-1}\sum_{l=0}^{i-1}s_{i,m,l}v_{i,m,l}\right)}{V_{i}}$$

### The population model

The PM tracks susceptible and resistant phenotypes over a sequence of time periods for a population subject to a given vector-control treatment. The key outputs, calculated for each time period, are the proportion of the population with resistant and susceptible phenotypes and the overall reduction in infectious bites across the population compared to a susceptible population with no vector-control treatment.

The variables and parameters for the PM are described in Table 3, and baseline values used in the analysis are summarized in Table 4.

**Table 3:** Variables and parameters for the population model

**Variable or Parameter**	**Symbol**	**Comments & Constraints**
Period number (periods over which the population is tracked)^*^	*n*	0<*n*
Dominance of resistance allele	*d*	dominant *d* = 1
		recessive *d* = 0
Genotype (normal allele s, resistant allele r)	*g*	(s,s) *g* = 1
		(s,r) *g* = 2
		(r,r) *g* = 3
Proportion of total population having genotype *g* at start of period *n*	*G*_*g,n*_	
Proportion of the population resistant at start of period *n*	*R*_*n*_	
Average number of infectious bites per mosquito in population in period *n*	*M*_*n*_	
Size of initial population (susceptibles in the presence of treatment) as proportion of base population (susceptibles without treatment)	*J*	*value from FCM*
Population size in period *n* as proportion of initial population size	*W*_*n*_	
Average infectious bites during one time period from an untreated population	*q*	*value from FCM*
Number of infectious bites from treated population during time period *n,* expressed as a % of the number of infectious bites during one time period from a susceptible population without treatment,	*Q*_*n*_	*Chosen measure of control*
Number of periods between egg-laying and adult emergence	*Φ*	*Input*

^*^As for the FCM, the duration of one gonotrophic cycle is used as a unit of time. For convenience we use ‘cycles’ to refer to mosquito age and ‘periods’ to refer to the sequential time periods for which values are calculated in the PM.

**Table 4:** Values used in the population model for this analysis

**Variable or Parameter**	**Symbol**	**Value**
Proportion of total population having genotype *g* at start of period 1	*G*_*g,1*_	*G*_*1,1*_*=* 1-*G*_*2,1*_
		*G*_*2,1*_*=* 10^-9^
		*G*_*3,1*_ = 0
Dominance of resistance allele *(0=recessive, 1=dominant)*	*d*	*d* = 1
Number of periods between egg-laying and adult emergence	*Φ*	3
Fitness factor for males with genotype *g*	*f*_*g*_	*f*_*1*_*=f*_*2*_*=f*_*3*_*=*1.00

All other input values use results calculated by the FCM.

A detailed derivation of the model is given in Additional file 2: Appendix B. In brief, the PM works in discrete time periods, each equivalent to the length of one gonotrophic cycle, with recruitment of newly emerged adult mosquitoes treated as occurring at the start of each time period. For each sequential time period, the proportion of the population comprised by each genotype in each age class is calculated, reflecting the genotypes of new adult recruits and the survival of adults in each age class from the preceding period. This is then used to calculate the proportion of the total population in time period *n* with homozygous recessive (*G*_*3,n*_) and heterozygous (*G*_*2,n*_) genotypes, from which *R*_*n*_ is calculated, the proportion of the population with a resistant phenotype in period *n*, with *R*_*n*_ = *G*_3,*n*_ + *G*_2,*n*_*d*. Dominance is actioned by the value of *d*, which is 0 when resistance is assumed recessive, and 1 when it is assumed to be dominant.

Results from the FCM are used by the PM to calculate the average number of infectious bites per mosquito in the population during each time period. From this *Q*_*n*_, the number of infectious bites given by the population as a whole relative to those given by an untreated population, can be calculated for each time period as $Q_{n}=\frac{M_{n}W_{n}J}{q}$.

### Assumptions

The model does not attempt to capture the effects of mutational processes or stochastic demographic effects on the origin and initial spread of very low numbers of resistance alleles, and so it is assumed that resistant phenotypes are already established at a low frequency in the population at the start of the analysis. Resistance involves a single gene and a simple dominant/recessive process. Moreover, it is assumed that the size and age structure of the population at the start of the PM analysis is that achieved after sustained use in a susceptible population of the insecticide being evaluated, that there is no immigration or emigration, and the proportion of each genotype in the new adults joining the population matches that in the eggs from which they originate. Density dependence is assumed to occur at the mosquito larval stage and the number of adult mosquitoes recruited to the population per unit of time remains constant.

All model parameters are age-independent, apart from background mortality rates and the action of age-linked pesticides, with incremental mortality from fungal biopesticide infection varying according to the number of days since infection. Conventional insecticides affecting a susceptible individual are assumed to be instantly fatal. Mosquitoes choose human hosts at random, and the model does not capture feedback between numbers of infectious bites and the proportion of human hosts with infectious malaria. Malaria-infected mosquitoes never become uninfected. All feeding cycles are of equal duration and mosquitoes bite once in each cycle. All eggs laid are of equal quality and viability. The analysis assumes that malaria infection produces no effects on behaviour, background mortality or fecundity in infected mosquitoes, and fungus-infected mosquitoes that survive and lay eggs are assumed to lay as many eggs at each laying event as uninfected individuals.

Mosquitoes are assumed to contact the chemical or biopesticide when resting after biting a human host, reflecting an application method essentially consistent with IRS. Avoidance behaviour such as outdoor feeding and outdoor resting is not reflected in the coverage values for susceptible mosquitoes since it comprises a method of resistance.

### Analysis

**Figure 2:**
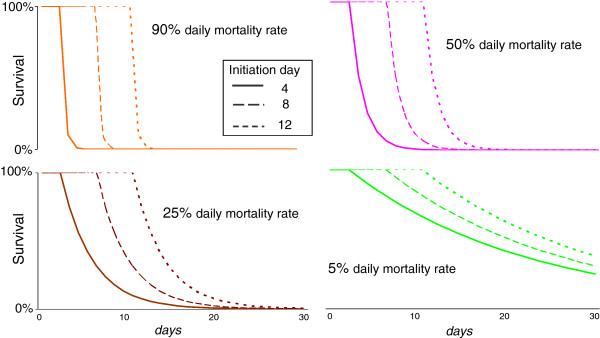
**Illustrative survival curves for a range of simple virulence mortality assumptions.** Survival curves illustrating mortalities defined by two simple virulence parameters. For each illustrated pair of values, mortality is zero until the specified initiation day, and is thereafter maintained at the indicated fixed daily mortality rate. Initiation day and mortality rate are the two parameters used to define the assumed incremental mortality generated by a given biopesticide infection.

A number of fungal strains have now been tested in laboratory mosquito populations, and a wide range of mortality characteristics have been observed around the basic pattern of initial fungal growth and development followed by an increase in observable mosquito mortality [11,15,16,18,37]. This suggests that most virulence profiles are potentially available, and in a search for generalizable results this analysis therefore uses highly simplified virulence mortality characteristics, defined by two parameters, ‘initiation day’, the time from infection to the onset of fungus-induced mortality, and the daily mortality rate from that point (Figure 2).

Fungal biopesticides can also impact mosquito feeding propensity and flight capacity in the days before mosquito death [11]. A mosquito which no longer attempts to feed or to lay eggs is effectively dead from the perspectives of fitness and disease transmission. For the purpose of the model therefore, ‘mortality’ encompasses cessation of feeding and reproduction, as well as actual death.

## Results

### Coverage and virulence

**Figure 3:**
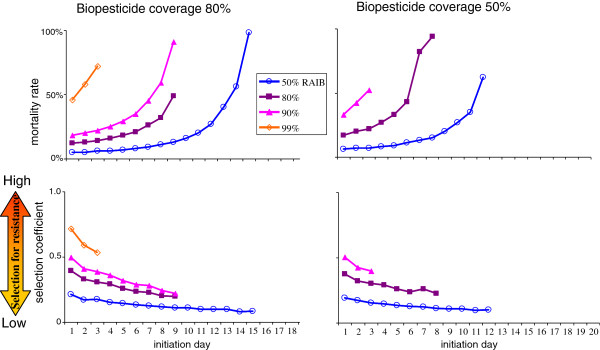
**Comparison of virulence characteristics and fitness costs associated with given reductions in EIR.** Top panels show different combinations of values for initiation day (x-axis) and daily mortality rate (y-axis) which achieve the denoted reductions in EIR (RAIB). The mortality rate required to achieve a given RAIB increases for later initiation days, up to an initiation day beyond which the target RAIB cannot be achieved, at which point the plots stop. With 50% biopesticide coverage, no virulence parameter combinations achieve 99% RAIB. Bottom panels show the selection coefficients corresponding to the same set of virulence parameter values, eg, the 99% RAIB value plotted for initiation day 2 gives the fitness cost for susceptibility to a biopesticide with initiation day 2 combined with the mortality rate required to achieve a 99% RAIB. Higher selection coefficients indicate stronger selection pressure for resistance.

The proportionate reduction in EIR generated by use of a biopesticide is affected by fungal virulence and coverage (Figure 3). For a given level of coverage, similar levels of EIR reduction are achieved by various combinations of the two parameters used to summarize virulence (initiation day and mortality rate (Figure 2)). Unsurprisingly, the longer a fungus takes to initiate mortality, the greater the subsequent mortality rate has to be to maintain a given level of reduction in EIR. There are limits to the EIR reductions that can be achieved at low virulence and/or low coverage.

For equivalent reductions in EIR, selection for resistance is best minimized by high coverage with late initiation day, high mortality rate biopesticides. For example, the lowest selection coefficient associated with a 90% RAIB at 80% coverage is 21%, with day 9 initiation and a 91% mortality rate. At 50% coverage the lowest selection coefficient available in combination with 90% RAIB is 40%.

**Figure 4:**
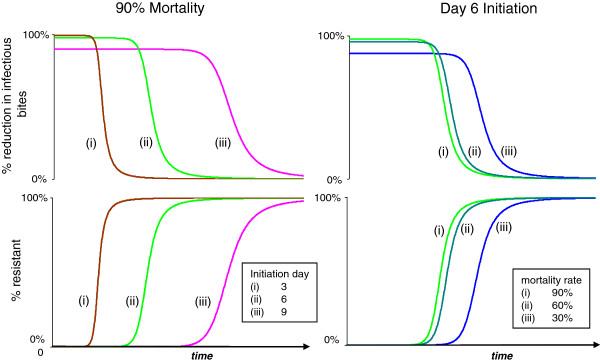
**Population level infectious bite rate and proportion resistant for populations exposed to different biopesticides.** Top panels show the population reduction in infectious bites per unit of time for each of five different virulence combinations, and the change in this value over time with the spread of resistance to the treatments, shown in bottom panels. 80% coverage assumed throughout.

The temporal dynamics of EIR reduction and resistance evolution are shown in Figure 4. Predictably, more virulent biopesticides give better population-level reductions in EIR to begin with, but they then drive the evolution of resistance more rapidly. The speed of resistance evolution is more sensitive to the timing of mortality onset than to the incremental mortality rate.

**Figure 5:**
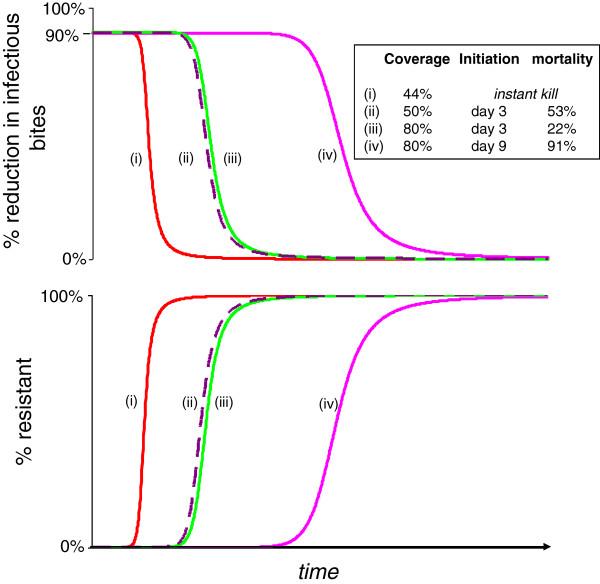
**Comparison of four interventions providing a 90% initial reduction in infectious bites.** Plots show the change over time in the proportion of resistant individuals (bottom panel) and the percentage reduction in population level infectious bites (top panel) for a mosquito population consistently exposed to one of four vector control treatments, all chosen to give the same 90% initial reduction in EIR.

The evolutionary dynamics and resulting pattern of control failure differ markedly for different insecticides even when they give identical reductions in EIR in the pre-evolutionary phase (Figure 5). Conventional instant-kill chemical insecticide (with coverage adjusted to achieve the same initial control) fails first. The longest time to product failure is offered by a fungal biopesticide with relatively late mortality initiation, which then kills at a very high rate (Figure 5).

**Figure 6:**
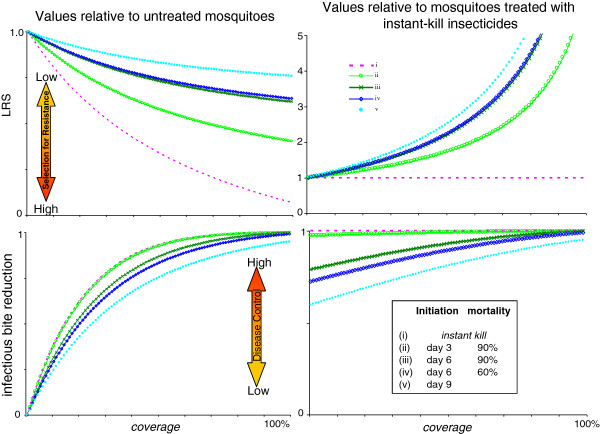
**Comparison of conventional instant-kill chemical insecticide and four biopesticides across a range of coverage values.** Lifetime reproductive success with interventions as a proportion of LRS for untreated mosquitoes (top left panel) and as a proportion of LRS for mosquitoes treated with an instant-kill insecticide (top right panel). Reduction in average infectious bites per mosquito lifetime with interventions, compared to the value for untreated mosquitoes (bottom left panel), 0 = no reduction in infectious bites, 1.00 = no infectious bites. Reduction in infectious bites with interventions *vs* untreated mosquitoes, compared to the reduction achieved using a conventional instant kill insecticide (bottom right panel), 1.00 means reduction equal to that achieved by instant-kill insecticide.

**Figure 7:**
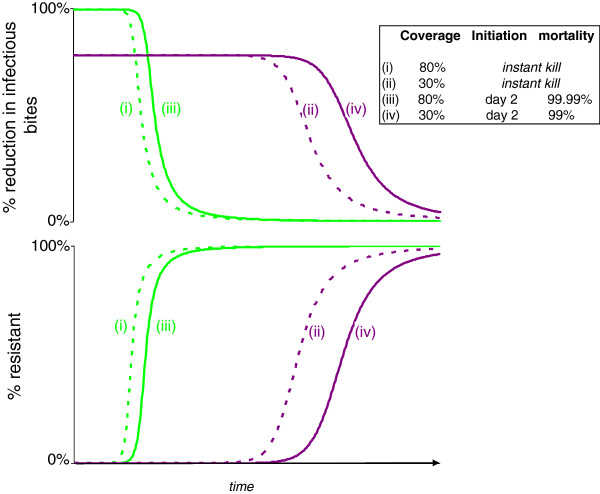
**Comparison of resistance spread and consequent increases in infectious bites with instant-kill and fungal biopesticides.** Biopesticide virulence selected to give pre-resistance EIR reduction matching instant-kill pesticides at 80% or 30% coverage. Plots show the proportion of the population with resistant phenotypes, and the corresponding values for population-level reduction in infectious bites per unit of time compared to an untreated population.

Clearly, the probability that a mosquito contacts and is affected by a vector-control treatment has a significant impact on both the reduction in EIR and reproductive success. Reductions in EIR improve as coverage is increased, but the strength of selection for resistance also increases (Figure 6, left panels). This illustrates the predictable trade-off between the best transmission control, obtained at high coverage, and the best resistance management, obtained at low coverage. When compared to the currently available alternative, a conventional instant-kill chemical insecticide, however, the relative values for EIR reduction and resistance management with the biopesticides are maximized at the high coverage values which correspond to the best transmission control and the strongest selection pressures for resistance (Figure 6, right panels). Even a biopesticide with sufficiently high virulence to match the initial EIR reduction of instant-kill insecticides at the same coverage levels offers some benefit in terms of useful life (Figure 7). This is because fungus-infected mosquitoes are still able to achieve some reproduction before being killed, thus somewhat reducing the selection for resistance.

### Repellency

**Figure 8:**
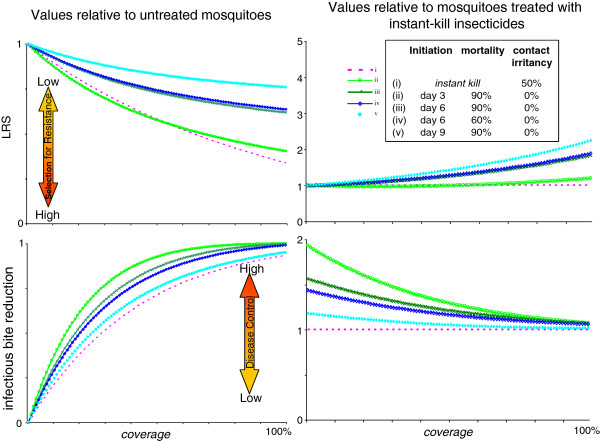
**Comparison between biopesticides and instant-kill insecticide with 50% contact irritancy, across range of coverage values.** Lifetime reproductive success with interventions as a proportion of LRS for untreated mosquitoes (top left panel) and as a proportion of LRS for mosquitoes treated with an instant-kill insecticide with 50% contact irritancy (top right panel). Reduction in average infectious bites per mosquito lifetimewith interventions, compared to the value for untreated mosquitoes (bottom left panel), 0 = no reduction in infectious bites, 1.00 = no infectious bites. Reduction in infectious bites with interventions *vs* untreated mosquitoes, compared to the reduction achieved using a conventional instant kill insecticide with 50% contact irritancy (bottom right panel), 1.00 means reduction in AIB equal to that achieved by instant-kill insecticide with 50% contact irritancy.

One of the most commonly used classes of conventional insecticides, pyrethroids, have high contact irritancy (also called excito-repellency), causing approximately 50% of mosquitoes contacting treated surfaces to be repelled without acquiring a harmful dose [23-25,38]. There is no indication of any repellency effects for the fungal biopesticides [26]. For IRS, if 50% of mosquitoes contacting the instant-kill insecticide are unaffected by it, then, for equivalent spray coverage, fungal biopesticides offer better reductions in EIR at all coverage levels, whilst maintaining selection benefits for all but the most virulent strain at the lowest coverage (Figure 8).

### Malaria interactions

**Figure 9:**
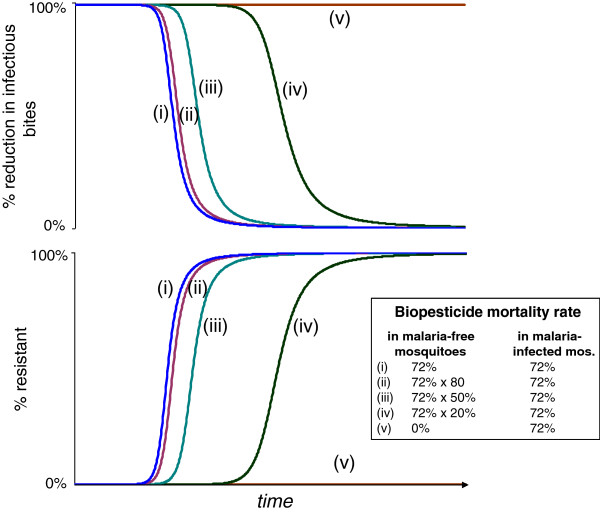
**Differential mortality in malaria-infected and malaria-free mosquitoes.** Comparison of speed of spread of resistance and consequent loss of transmission control for populations treated with one of five fungal biopesticides with differential mortality rates in malaria-infected mosquitoes. Plots show the proportion of the population with resistant phenotypes, and the corresponding values for population-level reductions in infectious bites per unit of time compared to an untreated population. The biopesticides all have day 3 initiation of a 72% daily mortality rate for malaria infected mosquitoes, giving an initial 99% reduction in infectious bites per time period.

Some fungal strains have been shown to have higher virulence in malaria-infected mosquitoes than in those without malaria infection [15]. The trade-off between reducing EIR and resistance management is greatly reduced where fungal virulence is lower in malaria-free mosquitoes, with selection for resistance virtually eliminated if the fungus induces mortality exclusively in malaria-infected mosquitoes (Figure 9).

## Discussion

**Figure 10:**
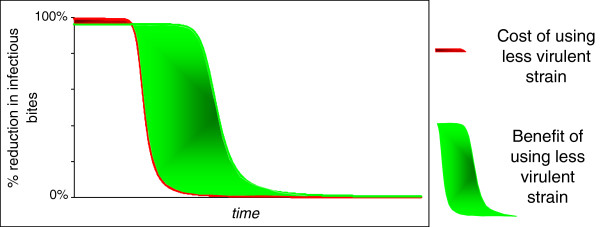
**Schematic illustration of the trade-off between initial levels of control and product useful life.** The green and red areas highlight the differences in infectious bites resulting from a choice between a high-virulence intervention with high initial infectious bite reduction and high selection for resistance *versus* a lower-virulence intervention with lower initial benefits and lower selection for resistance. The red area highlights the additional infectious bites associated with choosing the lower virulence option, before resistance spreads. The green area shows the additional infectious bites associated with choosing the high-virulence option in the long term once resistance spreads.

Variation in the virulence characteristics of potential biopesticides offers scope for selecting strains targeted to provide desirable combinations of reduced transmission and resistance management. A number of virulence phenotypes can provide equivalent levels of EIR reduction (Figure 3), and in general high biopesticide-induced mortality rates commencing as late as possible offer better resistance management for a given level of EIR reduction (Figures 3 and 5). There is nonetheless a trade-off between extending the time taken for resistance evolution to undermine efficacy of a pesticide, and the initial reductions in transmission (Figure 4). In general terms, more virulent fungal strains better reduce transmission initially, but at the cost of stronger selection for resistance, and consequently a shorter useful life (Figure 10).

Although high coverage offers scope to use less virulent fungal strains to reduce EIR, for given virulence parameters, higher levels of coverage also generate stronger selection for resistance, for both conventional and biopesticide interventions. Remembering that the biopesticides must be considered in relation to the best currently used approaches, it is interesting to note that in relative terms, the benefits of biopesticides *versus* conventional instant-kill insecticides are maximized at high coverage for both transmission control and resistance management (Figure 6).

The relative importance of initial control *versus* product lifespan depends on a large number of factors, including the availability of alternative replacement treatments, the meaning in terms of human morbidity and mortality of a smaller reduction in EIR at the outset, and the realities of public budgets and other resources. The relative costs and benefits also change if the biopesticide is being considered for use as part of a combination treatment with other interventions [34,39,40]. There is, therefore, no simple mathematical optimum for the many possible virulence schedules; the many possibilities need to be considered in context. In so far as it can be done without compromising transmission control, however, it is clearly beneficial to choose the biopesticide that generates the lowest selection for resistance in a particular context. For resistance management, the aim should be to achieve high levels of coverage, allowing less virulent fungal strains to achieve a given level of control, and maximizing their resistance management benefits over instant-kill insecticides.

Even strains sufficiently virulent to match the transmission-reducing characteristics of conventional instant-kill chemical insecticides at matching coverage levels still offer a small benefit in terms of the rate of spread of resistance (Figure 7). Such a resistance management gain would be enhanced by any fitness costs associated with resistance [8].

The conclusions presented here are independent of the method of resistance (e.g., metabolic or behavioural), provided resistance is genetically determined. It is assumed however that resistance is a binary quality, with mosquitoes either experiencing the full effects of a control measure, or remaining completely unaffected by it. The analysis of the speed of spread of resistance here thus assumes that susceptible mosquitoes experience infections with the specified virulence characteristics, and that resistant mosquitoes have no fungal mortality. In reality, it is more probable that a resistance/tolerance process would operate, with resistant mosquitoes still becoming infected, but experiencing a lower mortality rate than fully susceptible individuals. The spread of resistance would therefore effectively comprise a reduction in fungal virulence, rather than a complete loss of control. Considering the results presented in Figure 4, for example, this would mean that the spread of resistance to the highest virulence biopesticides, rather than comprising a steep function to complete resistance and total loss of transmission control, would move to the curves calculated for sequentially less virulent strains, as resistance converts high virulence strains to low virulence strains, offering even more beneficial resistance management possibilities. Future analyses could explore the impact of hypothetical resistance mechanisms that might operate with respect to conventional and fungal pesticides. The analyses presented here could also be extended to evaluate the impact of malaria infection on mosquito survival, fecundity and behaviour and variation in fecundity with mosquito age.

Certain widely used pyrethroid insecticides have high contact repellency, with studies suggesting that around 50% of mosquitoes landing on treated surfaces may leave before acquiring a fatal dose [23-25,38]. Whilst this potentially enhances the impact of pyrethroid-treated bed nets on transmission by deflecting mosquitoes away from protected humans before they bite, for IRS it results in mosquitoes surviving to potentially transmit malaria in later feeding cycles [24]. Thus, for this group of conventional insecticides, the composite ‘coverage’ value at a given level of spray cover, would be half that for biopesticides, and could never be greater than 50%. Comparing biopesticide performance with that of a conventional insecticide, and assuming 50% contact repellency (Figure 8) across a full range of coverage values, fungi better reduce transmission than pyrethroid IRS, while still maintaining some resistance management benefits. This suggests that, for all spray coverage values, suitably virulent fungal strains might provide a better option for IRS-based vector interventions than contact-repellent pyrethroids. If only low levels of spray coverage are achievable, replacing repellent pyrethroids with high-virulence fungal treatments could significantly improve the achievable EIR reduction, without significantly increasing selection for resistance, which is in any case relatively weak at low coverage (Figure 8). Where high spray coverage is achievable, replacing pyrethroids with relatively low-virulence fungal treatments could give improvements in both transmission control and resistance management, since the relative fitness of susceptible mosquitoes would be potentially doubled.

The analysis shows that in all cases, having higher fungal-induced mortality in malaria-infected mosquitoes than in uninfected mosquitoes minimizes the fitness costs associated with a given reduction in transmission (Figure 9). The ideal biopesticide from the resistance management perspective would be one that had little or no impact on mosquitoes not infected with malaria, but was strongly virulent in malaria-infected individuals. This might be possible since malaria infection can impose significant metabolic and immunological challenges to mosquitoes [41-44]. There is only a minimal trade-off between transmission control and resistance management in malaria-linked incremental biopesticide mortality. By changing the fitness cost to the mosquito of malaria infection, pesticides working in this way might also exert selection in favour of vector resistance to malaria, further enhancing the transmission-control benefits from the intervention. Strain selection or genetic modification should ideally target this trait. A further development of this principle would be fungal strains which specifically block development of the malaria parasite in the mosquito, or simply act as a delivery mechanism for anti-malaria interventions in the mosquito host (‘paratransgenesis’ [10,37]), with minimum survival or fecundity costs to the mosquito. It must be noted, however, that this potentially moves selection for resistance from the mosquito to the malaria parasite, which has so far proved extraordinarily adept at evolving its way out of trouble.

## Conclusions

This analysis shows that fungal biopesticides have the potential to significantly reduce EIR while imposing only weak selection for resistance. There is always a trade-off between the magnitude of the initial reductions in transmission and maintaining those reductions in the longer term. Given the severe human and economic consequences of malaria transmission, choosing an intervention which does not maximally reduce transmission at the outset requires very careful justification. However, the analyses presented here show that fungal biopesticides can offer equivalent or better reductions in transmission than existing interventions in both the short and long term. This is especially true where existing conventional chemical pesticides have high contact irritancy or resistance to them has already begun to spread. The theoretical analyses presented here should help define the vector mortality profiles required to maximize the sustained malaria control potential of fungal biopesticides, or indeed other novel biological or chemical insecticides.

## Abbreviations

AIB: Average number of infectious bites per mosquito per lifetime; EIR: Entomological inoculation rate, the number of infectious bites per person per unit of time; FCM: Feeding cycle model; IRS: Indoor residual spraying; LLA: Late-life acting; LRS: Lifetime reproductive success; PM: Population model; RAIB: Proportionate reduction in average number of infectious bites per mosquito per lifetime, compared to the number for untreated susceptible mosquitoes.

## Competing interests

The authors declare that they have no competing interests.

## Authors' contributions

PL developed and applied the model and contributed to study inception, interpretation of results and drafting of the manuscript. UG reviewed the model and contributed to drafting of the manuscript. MT and AR contributed to study inception, interpretation of results and drafting of the manuscript. All authors read and approved the final manuscript.

## Supplementary Material

Additional file 1 **Appendix A.** Derivation of the feeding cycle model. Detailed description of variables and calculations used in the feeding cycle model. Click here for file

Additional file 2 **Appendix B**. Derivation of the population model. Detailed description of variables and calculations used in the population model. Click here for file
