# Supplementary material for: Prospective malaria control using entomopathogenic fungi: comparative evaluation of impact on transmission and selection for resistance
Source: Malar J. 2012 Nov 22;11:383. doi: 10.1186/1475-2875-11-383 (PMC3519523; doi:10.1186/1475-2875-11-383)
Supplement: Additional file 1 — Appendix A. Derivation of the feeding cycle model. Detailed description of variables and calculations used in the feeding cycle model. [file 1475-2875-11-383-S1.pdf]

Appendix A

Derivation of the Feeding Cycle Model

The primary definitions for the FCM are given in the main text and Table 1. Full supporting details are given below and in Table 5.

The average number of eggs laid in cycle i, by mosquitoes starting cycle i with malaria status m and biopesticide status l is defined as

$$f_{i,m,l} = L(1-\theta)E_{1,m}E_{2,l}\left(\sum_{h=1}^{3} q_{i,m,l,h}\right)z_{i,m,l} \qquad l>0$$

$$f_{i,m,0} = L(1-\theta)E_{1,m}E_{2,0}\left(q_{i,m,l,1}z_{i,m,l} + \sum_{h=1}^{2} q_{i,m,l,h}\left(\left(1-X\right)z^{C}{}_{i,m} + Xz^{A}{}_{i,m}\right)\right)$$

The average probability of an adult mosquito surviving to the start of cycle i, V_i , is 1 for cycle 1. For all subsequent cycles, V_i is the sum for all possible combinations of fungus & malaria status of the probabilities of an adult mosquito surviving to the start of cycle i.

$$V_1 = 1$$

$$V_i = \sum_{m=0}^{i-1}\sum_{l=0}^{i-1} v_{i,m,l} \qquad i>1$$

The various survival probabilities, $v_{i,m,l}$ are calculated as follows. The average probability of an adult mosquito surviving to the start of cycle i, and being in the mth cycle of malaria infection and the lth cycle of fungus infection at the start of cycle i, $v_{i,m,l}$, is 1.00 at the start of cycle 1, and thereafter calculated for each possible combination of m and l at the start of the preceding cycle.

$$v_{1,0,0} = 1.00$$

The probability of surviving to the start of cycle i with no malaria or biopesticide infection, $v_{i,0,0}$, is the probability of surviving, uninfected, to the start of the previous cycle, and then surviving biting a non-human host, or biting a human host without being infected by malaria or biopesticide, and then surviving through laying.

$$v_{i,0,0} = v_{i-1,0,0}\left(q_{i-1,0,0,1} + \left(q_{i-1,0,0,2} + q_{i-1,0,0,3}\left(1-M\right)\right)\left(1-k_{i-1}\right)\left(1-X\right)\right)z^{C}_{i-1,0} \qquad i>1$$

The probability of surviving to the start of cycle *i* with newly acquired infections for malaria and biopesticide, $v_{i,1,1}$, is the probability of surviving, uninfected, to the start of the previous cycle, and then surviving biting an infectious human host, becoming infected with malaria and a biopesticide, and then surviving through laying, without being killed by any rapid biopesticide mortality.

$$v_{i,1,1} = v_{i-1,0,0} q_{i-1,0,0,3} \left(1 - k_{i-1}\right) MX \; z^{A}_{i-1,0} \qquad i > 1$$

The probability of surviving to the start of cycle *i* with a newly acquired malaria infection, and no new biopesticide infection, $v_{i,1,0}$, is the probability of surviving, uninfected, to the start of the previous cycle, and then surviving biting an infectious human host and becoming infected by malaria, not acquiring a biopesticide infection, and then surviving through laying.

$$v_{i,1,0} = v_{i-1,0,0} q_{i-1,0,0,3} M \left(1 - k_{i-1}\right)\left(1 - X\right) z^{C}_{i-1,0} \qquad i > 1$$

The probability of surviving to the start of cycle *i* with a newly acquired malaria infection, and an existing biopesticide infection, $v_{i,1,l}$ is the probability of surviving, with a biopesticide infection, but no malaria infection, to the start of the previous cycle, and then surviving biting an infectious human host and becoming infected by malaria and then surviving through laying, with survival probabilities reflecting additional mortality from the biopesticide infection.

$$v_{i,1,l} = v_{i-1,0,l-1} q_{i-1,0,l-1,3} \left(1 - k_{i-1}\right) M \; z_{i-1,0,l-1} \qquad i > 1 \;\; l > 1$$

The probability of surviving to the start of cycle *i* with an existing malaria infection, and no biopesticide infection, $v_{i,m,0}$, is the probability of surviving, with a malaria infection but no biopesticide infection, to the start of the previous cycle, and then surviving biting a non-human host or biting a human host without becoming infected by a biopesticide, and then surviving through laying.

$$v_{i,m,0} = v_{i-1,m-1,0} \left(q_{i-1,m-1,0,1} + \left(q_{i-1,m-1,0,2} + q_{i-1,m-1,0,3} \right) \left(1 - k_{i-1}\right)\left(1 - X\right) \right) z^{C}_{i-1,m-1}$$

$i > 1 \;\; m > 1$

The probability of surviving to the start of cycle *i* with no malaria infection, and a newly acquired biopesticide infection, $v_{i,0,1}$, is the probability of surviving, with no malaria or biopesticide infection, to the start of the previous cycle, and then surviving biting a human host, not acquiring a malaria infection and becoming infected by a biopesticide, and then surviving through laying, without being killed by any rapid biopesticide mortality.

$$v_{i,0,1} = v_{i-1,0,0} \left(q_{i-1,0,0,2} + q_{i-1,0,0,3} \left(1 - M\right) \right) \left(1 - k_{i-1}\right) X z^{A}_{i-1,0} \quad i > 1$$

The probability of surviving to the start of cycle *i* with an existing malaria infection, and a newly acquired biopesticide infection, $v_{i,m,l}$, is the probability of surviving, with a malaria infection but no biopesticide infection, to the start of the previous cycle, surviving biting a human host and becoming infected by a biopesticide, and then surviving through laying, without being killed by any rapid biopesticide mortality.

$$v_{i,m,1} = v_{i-1,m-1,0} \left(q_{i-1,m-1,0,2} + q_{i-1,m-1,0,3} \right) \left(1 - k_{i-1}\right) X \, z^{A}_{i-1,m-1} \qquad i > 1 \quad m > 1$$

The probability of surviving to the start of cycle *i* with no malaria infection, and an existing biopesticide infection, $v_{i,0,l}$, is the probability of surviving, with no malaria infection and an existing biopesticide infection, to the start of the previous cycle, and then surviving biting a

non-human host or biting a human host without acquiring a malaria infection, then surviving through laying, with survival probabilities reflecting additional mortality from the biopesticide infection.

$$v_{i,0,l} = v_{i-1,0,l-1}\left(q_{i-1,0,l-1,1} + \left(q_{i-1,0,l-1,2} + q_{i-1,0,l-1,3}\left(1-M\right)\right)\left(1-k_{i-1}\right)\right)z_{i-1,0,l-1} \quad i>1 \quad l>1$$

The probability of surviving to the start of cycle *i* with existing malaria and biopesticide infections, $v_{i,m,l}$, is the probability of surviving, with existing malaria and biopesticide infections, to the start of the previous cycle, surviving biting any host, then surviving through laying, with survival probabilities reflecting additional mortality from the biopesticide infection.

$$v_{i,m,l} = v_{i-1,m-1,l-1}\left(q_{i-1,m-1,l-1,1} + \left(q_{i-1,m-1,l-1,2} + q_{i-1,m-1,l-1,3}\right)\left(1-k_{i-1}\right)\right)z_{i-1,m-1,l-1} \quad i>1 \quad m>1 \quad l>1$$

The probabilities of surviving through cycle *i* are calculated as follows. The average probability, $s_{i,m,l}$, that mosquitoes starting cycle *i* with any malaria status and an existing biopesticide infection, will survive to the start of cycle *i* + 1 is calculated as the probability of surviving biting a non-human host, plus the probability of biting a human host without being killed by conventional instant-kill insecticides, and then surviving to lay.

$$s_{i,m,l} = \left(\left(1-k_i\right)\sum_{h=2}^{3} q_{i,m,l,h} + q_{i,m,l,1}\right)z_{i,m,l} \qquad l>0$$

The average probability, $s_{i,m,0}$, that mosquitoes starting cycle *i* with any malaria status and no biopesticide infection, will survive to the start of cycle *i* + 1 is calculated as the probability of surviving biting a non-human host, plus the probability of biting a human host without being killed by conventional instant-kill insecticides, and then either not acquiring a biopesticide infection, or acquiring a biopesticide infection but not being killed by the biopesticide before the end of the cycle, and then surviving to lay.

$$s_{i,m,0} = \left(\left(1-k_i\right)\sum_{h=2}^{3} q_{i,m,l,h}\left(\left(1-X\right)z_{i,m}^{C} + X\, z_{i,m}^{A}\right) + q_{i,m,l,1} z_{i.m}^{C}\right) \qquad i<\lambda$$

The probabilities of surviving host seeking and biting in cycle *i*, $q_{i,m,l,h}$, are calculated as follows. The probability, $q_{i,m,l,1}$ that a mosquito starting cycle *i* with malaria status *m,* and biopesticide status *l*, survives seeking and biting a non-human host, is the proportion of non-human hosts multiplied by the probability of surviving background mortality and the effects of any biopesticide infection whilst host seeking, and successfully biting without being killed whilst attacking host.

$$q_{i,m,l,1} = H\left(1-\sigma_{i,m,l}\right)e^{-b(r_{1,i}+\gamma_m+\alpha)}\left(1-a_1\right).$$

The probability, $q_{i,m,l,2}$, that a mosquito starting cycle *i* with malaria status *m,* and biopesticide status *l*, survives seeking and biting a human host not infectious for malaria is the proportion of hosts which are human and not infectious for malaria, multiplied by the probability of surviving background mortality and the effects of any biopesticide infection whilst host-seeking, and successfully biting without being killed whilst attacking host is

$$q_{i,m,l,2} = (1-p)(1-H)\left(1-\sigma_{i,m,l}\right)e^{-b(r_{1,i}+\gamma_m+\alpha)}(1-a_1).$$

The probability, $q_{i,m,l,3}$, that a mosquito starting cycle *i* with malaria status *m,* and biopesticide status *l*, survives seeking and biting a human host infectious for malaria is the proportion of hosts which are human and infectious for malaria, multiplied by the probability of surviving background mortality and the effects of any biopesticide infection whilst host seeking, and successfully biting without being killed whilst attacking host is

$$q_{i,m,l,3} = p(1-H)\left(1-\sigma_{i,m,l}\right)e^{-b(r_{1,i}+\gamma_m+\alpha)}(1-a_1).$$

The probabilities, $z_{i,m,l}$ that a mosquito starting cycle *i* with infection status *m,l*, will survive site seeking and lay eggs are calculated as follows.

$$z_{i,m,l} = (1-a_2)\left(1-\tau_{i,m,l}\right)e^{-\left(r_{3,i}\phi+r_{2,i}\eta+(\gamma_m+\alpha)(\phi+\eta)\right)} \qquad l>0$$

Probability, $z^A_{i,m}$ that a mosquito starting cycle *i* with no fungal infection, having survived biting a host, will survive site seeking and lay eggs if it acquires a new fungal infection during cycle *i* is

$$z^A_{i,m} = (1-a_2)\left(1-\tau_{i,m,0}\right)e^{-\left(r_{3,i}\phi+r_{2,i}\eta+(\gamma_m+\alpha)(\phi+\eta)\right)}.$$

Probability, $z^C_{i,m}$ that a mosquito starting cycle *i* with no fungal infection will survive site seeking and lay eggs if it does not acquire a new fungal infection is

$$z^C_{i,m} = (1-a_2)e^{-\left(r_{3,i}\phi+r_{2,i}\eta+(\gamma_m+\alpha)(\phi+\eta)\right)}.$$

The probabilities, $\sigma_{i,m,l}$, of dying from the effects of the biopesticide whilst host seeking in cycle *i*, for a mosquito starting cycle *i* with infection status *m*, *l*, are calculated as;

$$\sigma_{i,m,0} = 0$$

$$\sigma_{i,0,l} = 1-e^{-\left(\left(\lfloor wl-b\rfloor+1-(wl-b)\right)\beta_{(\lfloor wl-b+1\rfloor)}+\left(\sum_{n=\lfloor wl-b+1\rfloor+1}^{\lfloor wl\rfloor}\beta_n\right)+\left(wl-\lfloor wl\rfloor\right)\beta_{\lfloor wl+1\rfloor}\right)} \qquad l>0$$

$$\sigma_{i,m,l} = 1-e^{-\left(\left(\lfloor wl-b\rfloor+1-(wl-b)\right)\varepsilon_{(\lfloor wl-b\rfloor+1)}+\left(\sum_{n=\lfloor wl-b+1\rfloor+1}^{\lfloor wl\rfloor}\varepsilon_n\right)+\left(wl-\lfloor wl\rfloor\right)\varepsilon_{\lfloor wl+1\rfloor}\right)} \qquad l>0 \quad m>0$$

The probability, $\tau_{i,m,l}$, of dying from the action of the biopesticide between biting and laying during cycle *i*, having started the cycle with infection status *m,l* is calculated for mosquitoes which are already infected with fungus at the start of cycle *i*, or which become newly infected during cycle *i*, as follows;

$$\tau_{i,0,l} = 1 - e^{-\left(\left(\lfloor wl+1 \rfloor - wl \right) \beta_{\lfloor wl+1 \rfloor} + \left(\sum_{n=\lfloor wl+1 \rfloor +1}^{\lfloor wl+\phi+\eta \rfloor} \beta_n \right) + \left(wl+\phi+\eta - \lfloor wl+\phi+\eta \rfloor \right) \beta_{\lfloor wl+\phi+\eta+1 \rfloor} \right)} \qquad l > 0$$

$$\tau_{i,m,l} = 1 - e^{-\left(\left(\lfloor wl+1 \rfloor - wl \right) \varepsilon_{(\lfloor wl+1 \rfloor)} + \left(\sum_{n=\lfloor wl+1 \rfloor +1}^{\lfloor wl+\phi+\eta \rfloor} \varepsilon_n \right) + \left(wl+\phi+\eta - \lfloor wl+\phi+\eta \rfloor \right) \varepsilon_{\lfloor wl+\phi+\eta+1 \rfloor} \right)} \qquad l > 0 \quad m > 0$$

For mosquitoes newly infected during cycle *i*, the probabilities, $\tau_{i,m,0}$, of dying from the effects of the biopesticide before the end of the cycle are,

$$\tau_{i,0,0} = 1 - e^{-\left(\left(\sum_{n=1}^{\lfloor \phi+\eta \rfloor} \beta_n \right) + \left(\phi+\eta - \lfloor \phi+\eta \rfloor \right) \beta_{\lfloor \phi+\eta+1 \rfloor} \right)}$$

$$\tau_{i,m,0} = 1 - e^{-\left(\left(\sum_{n=1}^{\lfloor \phi+\eta \rfloor} \varepsilon_n \right) + \left(\phi+\eta - \lfloor \phi+\eta \rfloor \right) \varepsilon_{\lfloor \phi+\eta+1 \rfloor} \right)} \qquad m > 0$$

Table 5 Additional variables and parameters of the FCM

Variable or Parameter	Symbol	*Comments and Constraints*
Base instantaneous mortality rate per day for mosquito age *i* during activity B	$r_{B,i}$	*input*
Length of gonotrophic cycle (days)	w	*input*
Time spent host searching and feeding during a cycle (days)	b	*input*
Time spent finding oviposition site and laying during a cycle (days)	ϕ	*input*
Length of resting period (days)	η	*input*
Time required for parasite sporogonic development (days)	d	*input*
Proportion human population infectious for malaria	p	*input*
Probability attacks non-human host	H	*input*
Probability killed when attacking host before biting	a_1	*input*
Probability killed when attacking host after biting (excluding mortality from insecticide treatments)	a_2	*input*
Probability contacts and contracts biopesticide infection whilst resting after biting human host (biopesticide 'coverage')	X	*input*<br>*0 in cases not assuming use of biopesticide*
Probability becomes infected with malaria when biting infectious human host	M	*input*
Probability contacts and is killed by instant action conventional insecticide when attacking human host, after biting (conventional chemo 'coverage')	k_i	*input*<br>*0 in cases not assuming use of conventional insecticide*
Normalised number of eggs laid per successfully laying mosquito per cycle	L	*input*
Malaria-fecundity adjustment factor, proportionate number of eggs produced by mosquitoes with malaria infection age *m*	$E_{1,m}$	*input*
Biopesticide-fecundity adjustment factor, proportionate number of eggs produced by mosquitoes with biopesticide infection age *l*	$E_{2,l}$	*input*
Probability that a mosquito alive at start of cycle *i* with malaria status *m* and biopesticide status *l*, having survived to bite, then survives to lay eggs	$z_{i,m,l,}$	$m<i \quad l<i$
Instantaneous daily mortality rate from biopesticide on *n*th day after infection, for mosquitoes with no malaria infection	β_n	*input*
Instantaneous per day mortality rate from biopesticide on *n*th day after infection, for mosquitoes with malaria infection	ε_n	*input*
Incremental daily mortality rate with malaria infection age *m*	γ_m	*input*
Incremental daily mortality rate assumed as cost of resistance*	α	*input*
% reduction in egg production assumed as cost of resistance*	θ	*input*
Activity type, searching for host, resting, searching for laying site	B	*host-seeking = 1*<br>*resting = 2*<br>*site-seeking = 3*
Probability of dying from action of biopesticide before biting host in cycle *i*, for mosquito starting cycle *i* with malaria status *m* and biopesticide status *l*	$\sigma_{i,m,l}$	
Probability of dying from action of biopesticide between biting host and laying, in cycle *i*, for mosquito starting cycle *i* with malaria status *m* and biopesticide status *l*	$\tau_{i,m,l}$	$l>0$
Probability of dying from action of biopesticide between biting host and laying, in cycle *i*, for mosquito starting cycle *i* with malaria status *m* and biopesticide status 0 and acquiring a new biopesticide infection during the cycle	$\tau_{i,m,0}$	
Largest integer less than x	$\lfloor x \rfloor$	

* *Cost of resistance has not been used for the analysis in the current paper, but is an important element of previous analyses conducted with the model, focussing on theoretical chemical LLAs ([8])*
