# Supplementary material for: Prospective malaria control using entomopathogenic fungi: comparative evaluation of impact on transmission and selection for resistance
Source: Malar J. 2012 Nov 22;11:383. doi: 10.1186/1475-2875-11-383 (PMC3519523; doi:10.1186/1475-2875-11-383)
Supplement: Additional file 2 — Appendix B. Derivation of the population model. Detailed description of variables and calculations used in the population model. [file 1475-2875-11-383-S2.pdf]

Appendix B

Derivation of the population model

The primary definitions for the PM are given in the main text and Table 3. Full supporting details are given below and in Table 6.

The proportion of the population having genotype *g* at the start of period *n* is calculated as the part of the population with that genotype at the beginning of the period, expressed as a proportion of the initial population size, divided by the total population size at the start of period *n*, likewise expressed as a proportion of the initial population size.

$$G_{g,n} = \frac{Y_{g,n}}{W_n}$$

The part of the population having genotype *g* at the start of period *n*, $Y_{g,n}$, is calculated as the part of the population with that genotype at the beginning of the previous period multiplied by the proportion of such mosquitoes surviving to the end of the period, plus the new adults recruited at the start of period *n* with genotype *g*, all expressed as a proportion of the initial population size. $Y_{2,1} = G_{2,1}$ and $Y_{3,1} = G_{3,1}$.

$Y_{1,1} = 1 - Y_{2,1} - Y_{3,1}$ and

$$Y_{g,n} = Y_{g,n-1} P_{g,n-1} + KN_{g,n} \quad \text{with } n>1$$

The three genotypes map to the two phenotypes, resistant, *j*=2, and non-resistant, *j*=1, as follows;

$$g = 1 \rightarrow j = 1$$
$$g = 2 \rightarrow j = 1 + d$$
$$g = 3 \rightarrow j = 2$$

The proportion, $P_{g,n}$, of mosquitoes starting period *n* with genotype *g* which survive to the start of period *n*+1, is calculated as the sum of survival probabilities for mosquitoes with the phenotype generated by genotype *g,* in each age group multiplied by the proportion of the mosquitoes with genotype *g* in that age group.

$$P_{g,n} = \sum_{i=1}^{\lambda-1} C_{g,n,i} S_{j,i}$$

W_n, the total population size in period n, expressed as a proportion of the initial population size, is calculated as the total of population sizes for each genotype, expressed on the same basis.

$$W_n = \sum_{g=1}^{3} Y_{g,n}$$

The proportion, $N_{g,n}$, of new adults joining the population with a given phenotype in a given period is equal to the proportion of the genotype among the eggs giving rise to the new adults.

$$N_{g,n} = E_{g,n-\Phi} \quad n > \Phi$$

$$N_{g,n} = E_{g,1} \quad n \le \Phi$$

The average number of bites per mosquito in the population, M_n, is calculated as the sum of the totals for each genotype of the number of infectious bites per mosquito in each age category for mosquitoes with the applicable phenotype, multiplied by the proportion of mosquitoes with that genotype falling into each age category.

$$M_n = \sum_{g=1}^{3} \left(G_{g,n} \sum_{i=1}^{\lambda} C_{g,n,i} I_{j,i} \right)$$

The PM starts with a sub-population age-structure for each genotype within the whole population, $C_{g,1,i}$, reflecting the survival values generated in the FCM for mosquitoes subject to the appropriate treatment regime, with any relevant cost-of-resistance parameters.

In subsequent periods, the proportion of genotype *g* mosquitoes in age cycle 1 is calculated as the proportion of new adults with genotype *g* multiplied by the total number of new adults expressed as a proportion of the initial population size, divided by the total mosquitoes with genotype *g* at the start of period *n*, also expressed as a proportion of the initial population size

$$C_{g,n,1} = \frac{N_{g,n} K}{N_{g,n} K + Y_{g,n-1} P_{g,n-1}} \quad n > 1$$

For older age classes, the proportion of genotype *g* mosquitoes in each age cycle, $C_{g,n,i}$ is calculated as the proportion of mosquitoes with genotype *g* falling into the preceding age category multiplied by the relevant survival probability, divided by the total mosquitoes with genotype *g* at the start of period *n*

$$C_{g,n,i} = \frac{Y_{g,n-1} C_{g,n-1,i-1} S_{j,i-1}}{N_{g,n} K + Y_{g,n-1} P_{g,n-1}} \quad 1 < i \le \quad n > 1$$

The proportion $E_{g,n}$, of eggs produced in period *n* which have genotype *g*, is calculated as the number of eggs produced in period *n* with genotype *g*, divided by the total number of eggs produced in period *n*.

$$E_{g,n} = \frac{B_{g,n}}{B_{1,n} + B_{2,n} + B_{3,n}}$$

The numbers of eggs produced with each of the three possible genotypes, $B_{g,n}$, are calculated using the appropriate number of eggs per female for relevant phenotypes and ages, using the appropriate proportion of each genotype in the population to calculate the relevant allele

contribution from the female population, multiplied by allele proportions appropriate to the male population in the period of mating for each female age class.

$$B_{1,n} = \sum_{i=1}^{\lambda} \left((F_{1,i} C_{1,n,i} G_{1,n} + 0.5 F_{1+d,i} C_{2,n,i} G_{2,n}) A_{1,n-i+1} \right)$$

$$B_{2,n} = \sum_{i=1}^{\lambda} \left(F_{1,i} C_{1,n,i} G_{1,n} A_{2,n+1-i} + 0.5 F_{1+d,i} C_{2,n,i} G_{2,n} + F_{2,i} C_{3,n,i} G_{3,n} A_{1,n+1-i} \right)$$

$$B_{3,n} = \sum_{i=1}^{\lambda} \left((F_{2,i} C_{3,n,i} G_{3,n} + 0.5 F_{1+d,i} C_{2,n,i} G_{2,n}) A_{2,n+1-i} \right)$$

The proportions, $A_{1,n}$ and $A_{2,n}$ of susceptible and resistant alleles available from the male population in period n is calculated based on the proportion of newly hatched males with each genotype, and any relative fitness adjustments applied to different phenotypes. Male genotypes for matings prior to the start of the modelled time period are assumed to be consistent with those in the population in the first modelled period, so $A_{j,n} = A_{j,1} \quad n < 1$

$$A_{1,n} = (0.5 f_2 N_{2,n} + f_1 N_{1,n}) / (f_1 N_{1,n} + f_2 N_{2,n} + f_3 N_{3,n})$$

$$A_{2,n} = (0.5 f_2 N_{2,n} + f_3 N_{3,n}) / (f_1 N_{1,n} + f_2 N_{2,n} + f_3 N_{3,n})$$

Table 6

**Variable or Parameter**	**Symbol**	**Comments & Constraints**
Number of mosquito age classes included in analysis	λ	
Mosquito age (gonotrophic cycles)	i	$0 < i \le \lambda$
Phenotype	j	susceptible $j = 1$<br>resistant $j = 2$
Probability of survival for mosquitoes with phenotype *j*, to age *i*+1 from age *i* (*i*>=1)	$S_{j,i}$	*values from FCM*
Mosquitoes with genotype g at start of period n as percentage of initial population	Y_{gn}	
Allele *a* as proportion alleles contributed by male population in period *n*	$A_{a,n}$	$s \quad a = 1$<br>$r \quad a = 2$
Proportion of mosquitoes with genotype *g* which survive from start of period *n* to start of period *n*+1	$P_{g,n}$	
Proportion of mosquitoes with genotype *g* which are age *i* at start of period *n*	$C_{g,n,i}$	
Average number of eggs laid by females of phenotype *j*, aged *i*	$F_{j,i}$	*values from FCM*
Total number of eggs with genotype *g* laid in period *n*	$B_{g,n}$	
Proportion of all eggs laid in period *n* having genotype *g*	$E_{g,n}$	
Proportion of all new adults having genotype *g* at start of period *n*	$N_{g,n}$	$N_{2,1}$=$G_{2,1}$
Fitness factor for males with genotype *g*	f_g	
Average number of infectious bites per mosquito of phenotype j aged *i* in period *n*	$I_{j,i}$	*values from FCM*
**New adults as % initial population**	K	*values from FCM*
